# Reproduction of molecular subtypes of gastric adenocarcinoma by transcriptome sequencing of archival tissue

**DOI:** 10.1038/s41598-019-46216-6

**Published:** 2019-07-04

**Authors:** You Jeong Heo, Charny Park, Doyeong Yu, Jeeyun Lee, Kyoung-Mee Kim

**Affiliations:** 1 Department of Health Sciences and Technology, Samsung Advanced Institute for Health Sciences and Technology (SAIHST), Sungkyunkwan University and Samsung Medical Center, Seoul, Korea; 2 Department of Pathology and Translational Genomics, Samsung Medical Center, Sungkyunkwan University School of Medicine, Seoul, Korea; 3 Clinical Genome Analysis and Precision Medicine Branch, Research Institute, National Cancer Center, Goyang, Republic of Korea; 4 Department of Medicine, Division of Hematology-Oncology, Samsung Medical Center, Sungkyunkwan University School of Medicine, Seoul, Korea

**Keywords:** Molecular medicine, Gastroenterology

## Abstract

Gastric cancer (GC) is a heterogeneous disease, so molecular classification is important for selecting the most appropriate treatment strategies for GC patients. To be applicable in the clinic, there is an urgent need for a platform that will allow screening real-life archival tissue specimens. For this purpose, we performed RNA sequencing of 50 samples from our Asian Cancer Research Group (ACRG) GC cohort to reproduce the molecular subtypes of GC using archival tissues with different platforms. We filtered out genes from the epithelial-to-mesenchymal transition (EMT) and microsatellite instability-high (MSI) signatures (coefficient ≤ 0.4) followed by the ACRG molecular subtype strategy. Overall accuracy of reproduction of ACRG subtype was 66% (33/50). Given the importance of EMT subtype in future clinical trials, we further developed the minimum number of genes (10 genes) for EMT signatures correlating highly with the original EMT signatures (correlation ≥ 0.65). Using our 10-gene model, we could classify EMT subtypes with high sensitivity (0.9576) and specificity (0.811). In conclusion, we reproduced ACRG GC subtypes using different platforms and could predict EMT subtypes with 10 genes and are now planning to use them in our prospective clinical study of precision oncology in GC.

## Introduction

Gastric cancer (GC) is a heterogeneous disease with two distinct morphologic subtypes by Lauren’s classification (intestinal type and diffuse type) and with variable environmental etiologies, clinical manifestations, and genetic backgrounds. However, histological classifications of GC have limited clinical utility in the management of advanced diseases^1^. Genomic and transcriptomic analyses have defined the heterogeneity within the cancers and classified them into molecular subtypes characterized by specific genetic aberrations and expression signatures that suggest important biological differences^2^. A comprehensive study by The Cancer Genome Atlas (TCGA) consortium reported four molecular subtypes of GC: chromosomal instability (CIN), microsatellite instability-high (MSI), genomically stable **(**GS**)**, and Epstein-Barr virus (EBV) molecular subtypes^3^. However, the clinical significance of these subtypes is limited.

The Asian Cancer Research Group (ACRG) reported four molecular subtypes of GC with clinical significance based on mRNA expression profiles: microsatellite-stable (MSS)/TP53−, MSS/TP53+, MSI, and epithelial-to-mesenchymal transition (EMT) subtypes. In this molecular classification, the MSI subtype was consistently associated with favorable prognosis, while EMT GCs showed a significantly higher recurrence rate, higher probability of peritoneal seeding at recurrence, younger age at presentation, and poorer survival compared to other subtypes^4^. Hence, more aggressive treatment should be developed to improve survival^5^, although resistance to standard chemotherapy has also been reported for this subset of GC^6^.

The recent success of immunotherapies provides some evidence that additional approaches to genomic-driven precision medicine may be rewarding^7^. Checkpoint inhibitors has demonstrated that reactivating anti-tumor immune responses can regress cancers^8^. However, this only occurs in a fraction of patients and GCs are no exception. Recently, we reported that patients with EBV or MSI GC subtypes responded dramatically to pembrolizumab with overall response rates (ORR) of 100% and 85.7%, respectively^9^. However, patients with EMT subtypes showed no remarkable benefit from checkpoint blockade. These observations demonstrate the importance of molecular classification of GCs in the clinic and the need for a platform that will allow the screening of real-life biospecimens^7^. To molecularly characterize metastatic GC, development of technologies dedicated to archival tissue samples is urgently needed. In the present study, we performed RNA sequencing of 50 samples from an ACRG GC cohort to reproduce molecular classification in formalin-fixed paraffin-embedded (FFPE) tissue samples with different platforms that can be used as screening tools in the real world.

## Results

### Preprocessing of Gene Expression

**Figure 1:**
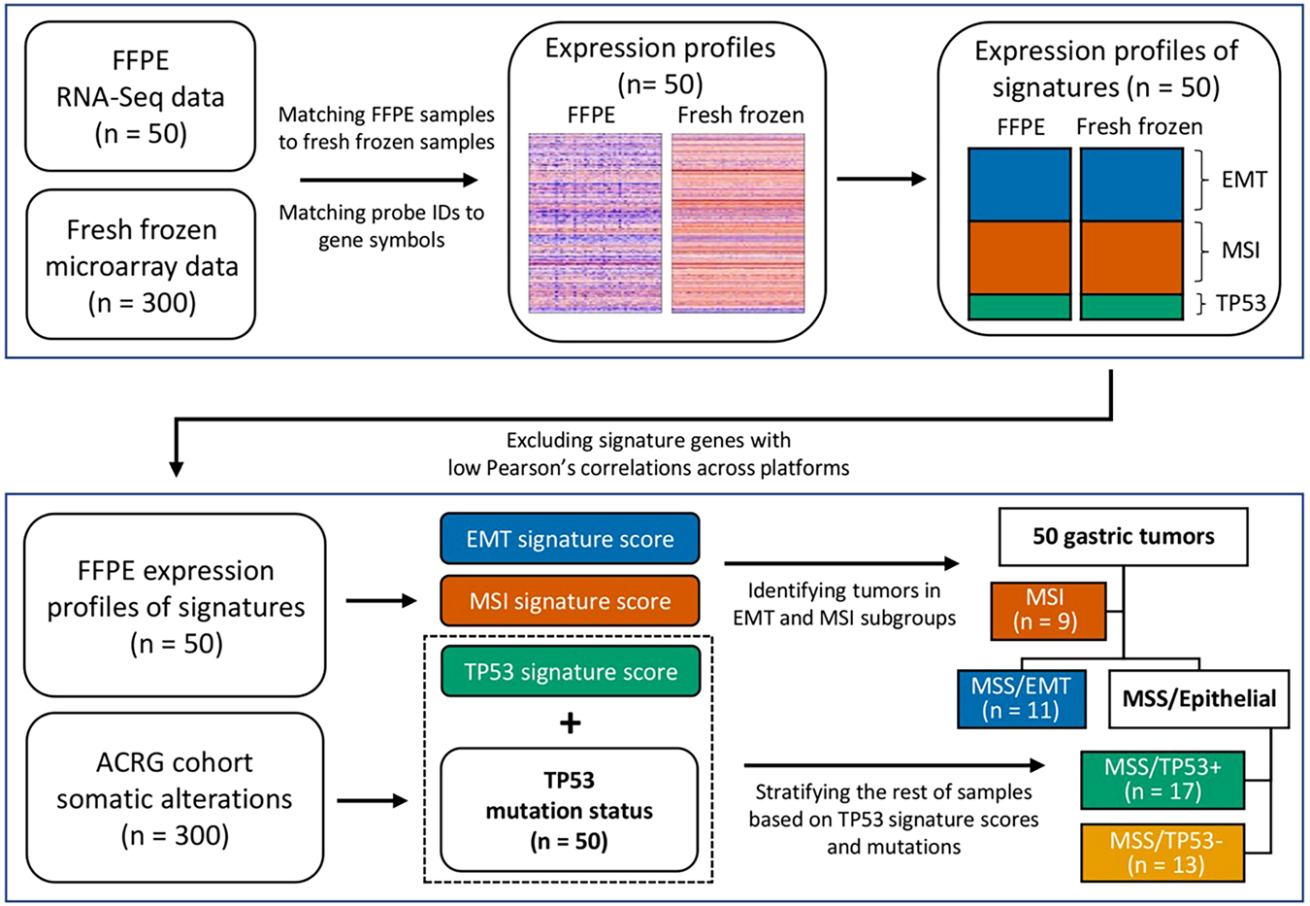
Analysis workflow to identify molecular subtype using 50 FFPE samples. Before subtype classification, we performed additional preprocessing, signature gene quality checks, and filtering. Next, we identified four subgroups by following a step-wise manner of ACRG approaches.

To achieve molecular subtype identification with different platforms, we randomly selected 50 FFPE tissues from the corresponding ACRG GC cohort^4^ and generated RNA transcriptome profiles. Before subtype identification, we performed preprocessing to compare signature gene expression statuses between two platforms and to validate signature scores. Highly correlated signature gene lists and signature score cutoffs were adjusted for new expression values originated from RNA sequencing (RNA-Seq). The overall process flow is shown in Fig. 1.

Before comparing gene expression profiles between RNA expression microarrays and RNA-Seqs, we constructed a gene-level expression for each platform by preprocessing and filtering. Microarray intensity was arranged into the gene expression profile by preprocessing to filter out low-expression genes and to rescale gene expression. Using microarray gene expression, we matched 45,782 of the 54,675 Affymetrix probe sets to 24,442 genes, among which 18,390 genes were present on both the microarray and RNA-Seq platforms. In the RNA-Seq platform, we estimated gene expression measure as Transcript Per Million (TPM) based on previously defined target regions and removed 5,424 genes with an average TPM below 2 based on RNA-Seq expression profiles, leaving 12,966 genes total.

Using the processed expression profiles, we investigated the overall status of the RNA-Seq data by comparing gene expression levels across all FFPE samples. Total gene expression profiles of FFPE samples exhibited similar distributions of log2 gene expression (Fig. S1). Also, we computed correlation coefficients for each matched pair of samples from the two platforms based on total gene expression profiles. The median Pearson and Spearman sample correlations were 0.54 and 0.52, respectively (Fig. S2).

**Figure 2:**
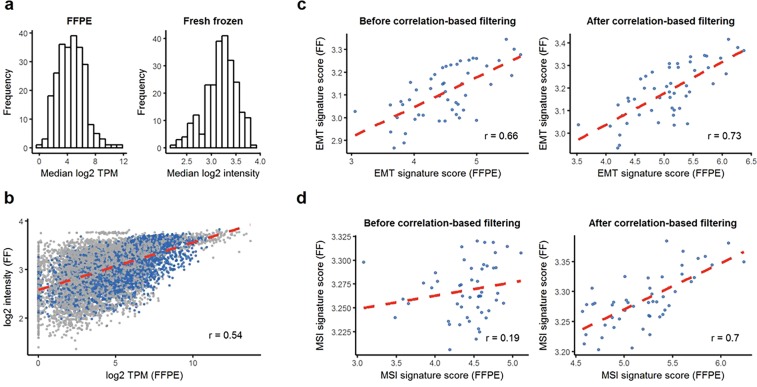
Overall gene expression and comparison between fresh frozen (FF) (Microarray) and FFPE (RNA-Seq). (**a**) Histogram of log2 expression values of signature genes in FF and FFPE. (**b**) Paired log expression value scatter plots across all sample and signature genes. Gene expression values that pass correlation filtering are indicated as blue dots. (**c**) EMT signature scores for FF and FFPE before/after filtering. (**d**) EMT signature scores for FF and FFPE before/after filtering.

We further examined the reproducibility between microarray and RNA-Seq with the expression signatures of EMT^10^, MSI^11^, and TP53 activity^12^, which were used for molecular subtype classification in the previous study^4^. The histograms show the distributions of the median log2 gene expression levels in the RNA-Seq and microarray data (Fig. 2a). While most of the signature genes in the microarray data had median log2 gene expression in the range of 2 to 4, the distribution of the RNA-Seq data was positively skewed with a wide range of expression. Figure 2b shows paired log2 expression values across all samples and signature genes. There were a number of pairs with zero log2 gene expression from the RNA-Seq data and robust signal intensity from the microarray data.

Additionally, we calculated correlation for each signature gene and each signature score. The median Pearson correlations for genes in the EMT and MSI signatures were 0.39 and 0.28, respectively (Fig. S3). The Pearson correlations for EMT and MSI signature scores between two platforms were 0.66 and 0.19, respectively (Fig. 2c,d, left). The TP53 signature is composed of two genes CDKN1A and MDM2, for which the Pearson correlations between two platforms were 0.54 and 0.33, respectively (Fig. S4).

### Reproduction of molecular subtypes of gastric adenocarcinomas

We identified molecular subtypes of 50 GC tumor samples based on FFPE RNA expression profiles via the ACRG classification approach. Prior to subtype classification, we filtered out genes with a Pearson correlation coefficient across the platforms lower than 0.4 from the EMT and MSI signatures. Finally, we could select 55 of 118 genes for EMT signature and 24 of 120 genes for MSI signature. We then carried out correlation computations again. The correlation for EMT signature scores increased to 0.73 (Fig. 2c, right). The Pearson correlation coefficient for MSI signature scores also dramatically increased to 0.7 (Fig. 2d right).

**Figure 3:**
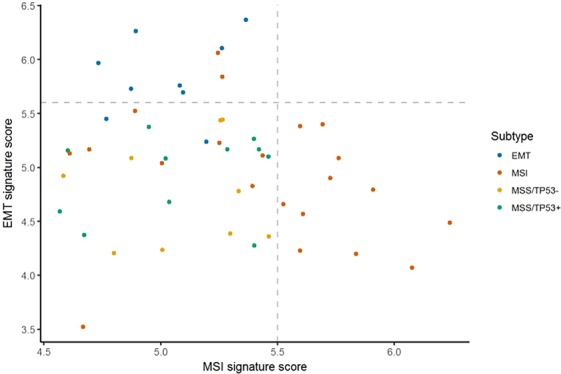
Score distribution of EMT and MSI signatures to assign mesenchyme and MSI subtypes, respectively.

Afterwards, we followed classification methods with the ACRG molecular subtype strategy in a step-wise manner. A scatterplot of EMT and MSI signature scores computed after the correlation-based selection is depicted in Fig. 3. We first assigned samples with EMT signature scores greater than 5.6 and MSI signature scores greater than 5.5 into EMT and MSI subtypes, respectively. Based on the ROC curve of a TP53 signature score for TP53 mutation, we found a threshold of 6.19 where the Youden’s index was maximized. We used the threshold to stratify the remaining samples into MSS/TP53+ and MSS/TP53- subtypes.

### Performance of new molecular classification

We defined the molecular subtypes by ACRG RNA expression profiling results with fresh frozen GC and paired normal gastric tissues as a gold standard and evaluated the reproducibility of the ACRG classification approach in FFPE samples. Overall accuracy was 66% (33/50) and Cohen’s kappa was 0.55. Overall accuracy was significantly higher than no information rate, which is the percentage of the largest class in the data (*P* = 3.9e-6). Sensitivity and specificity were computed for each subtype (Table 1). Despite poor sensitivity for identifying MSS/epithelial subtypes (MSS/TP53+ and MSS/TP53−), the molecular classification of FFPE samples demonstrated reasonable sensitivity and specificity for the MSI and EMT subtypes.

**Table 1:** Sensitivity and specificity by molecular subtype classification.

	EMT	MSI	MSS/TP53+	MSS/TP53−
Sensitivity (%) Specificity (%)	77.8 95.1	100 74.4	52.9 93.9	46.2 91.9

**Figure 4:**
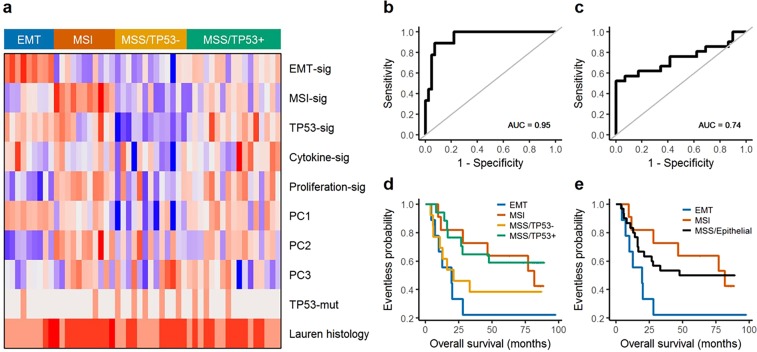
(**a**) Association of re-assigned subtypes with signatures, principal components, and clinical covariates. (**b**) ROC curve of EMT signature scores for EMT subtypes identified in a previous study. (**c**) ROC curve of MSI signature scores for MSI subtypes identified in a previous study. (**d**,**e**) Survival curves for re-classified subgroups. Log-rank test survival p-values were 0.148 (**d**) and 0.163 (**e**).

The heatmap shows the association of the re-assigned subtypes with the main three principal components (PC1–PC3), expression signature scores, TP53 mutation, and Lauren histology (Fig. 4a). We also estimated the ROC curves of EMT and MSI signature scores for the EMT and MSI subtypes identified in a previous study (Fig. 4b,c)^4^. The AUCs were 0.74 and 0.95, respectively, with both showing a significant association with the EMT and MSI subtypes (EMT: *P* = 1.4e-6, MSI: *P* = 0.003). We then applied the analytical procedures conducted in the previous ACRG cohort study^4^ to the expression profiles of RNA obtained from FFPE tissues and merged them with clinical information. We performed principal component analysis (PCA) for the exploratory analysis of expression profiles across re-assigned subtypes. We also tested the Spearman correlation between expression signature scores and PC1–PC3. PC1 had a significant correlation with EMT (0.62, *P* = 2.5e-6) and TP53 (0.66, *P* = 5.1e-7) signature scores. PC2 had a significant correlation with EMT (−0.66, *P* = 4.1e-7), MSI (0.62, *P* = 3.3e-6), and cell proliferation^13^ (0.74, *P* < 1e-10) signature scores. PC3 was significantly correlated with cytokine signaling scores^14^ (−0.76, *P* < 1e-10). In addition, survival analysis was conducted to compare overall survival among the reproduced new subtypes from ACRG data (Fig. 4d,e). We observed a trend towards superior survival for the MSI subtype, followed by the MSS/epithelial subtypes. The EMT subtype showed the worst prognosis.

### Development of prediction scores with 10 genes to discriminate EMT subtype

**Figure 5:**
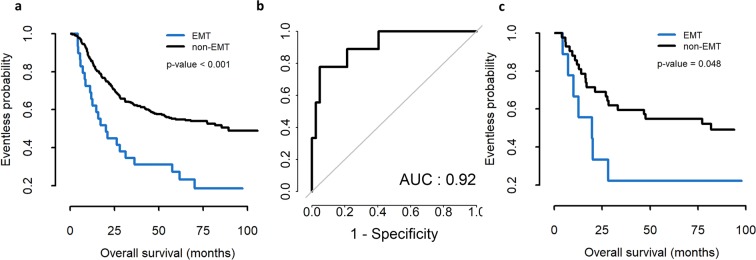
(**a**) Survival difference in patients with gastric carcinoma with EMT and non-EMT subtypes separated by prediction model with 10 genes in the ACRG cohort using fresh tissue specimens and calculated by the ROCR package. (**b**) The prediction of EMT subtype by 10-gene prediction model in the ACRG group. The sensitivity was 0.957 and the specificity was 0.811, with AUC of 0.92. (**c**) Survival differences in the present 50 RNA-Seq data with archival tissue samples.

Given the importance of EMT subtype in the future clinical trial design, we aimed to develop a minimum number of gene sets necessary to distinguish EMT subtypes using archival tissue to be applicable in the clinic. As PC2 had a significant correlation with EMT, we selected 10 genes showing high correlation (ranging from 0.61353 to 0.75455) with the ACRG EMT subtype obtained from expression arrays using FF tissue specimens and calculated new cutoff values using the ROCR package. This 10-gene model predicted survival difference significantly (*P* < 0.001) and the sensitivity and specificity to predict EMT subtype in the ACRG group were 0.957 and 0.811, respectively, with AUC (area under the ROC curve) at 0.92 (Fig. 5a,b). To validate EMT subtype obtained from the 10-gene prediction model, we used our previous mRNA expression results obtained using a different platform, the NanoString nCounter (NanoString Technologies, Seattle, WA), in archival FFPE tissues from ACRG cohorts (n = 71) and ARTIST cohorts (n = 72) and the results were comparable to our previous study with a 71-gene EMT signature (Fig. S5)^5^.

## Discussion

Great progress has been made toward the identification of molecular GC subtypes by gene expression profiling, which shows great promise for improving prognoses and identifying more appropriate therapies^15^. For a GC clinical trial to successfully use molecularly targeted agents or immunotherapy, it is crucial to discriminate EMT or MSI subtypes to enhance treatment outcome^5^. In the present study, we aimed to reproduce molecular subtypes of GC using archival tissue samples that are comparable to the results of RNA expression microarrays.

GC is traditionally divided into intestinal and diffuse histologic subtypes by Lauren’s classification, but recent molecular analyses have led to new molecular classifications based on genomic alterations^3^ or gene expression profiles^4,16^. The technology required for comprehensive molecular analyses is based on highly complex methodology and currently should not be replicated in standard laboratories lacking advanced instrumentation^17^. Therefore, many attempts towards simplification are ongoing, although results may not fully capture the underpinning complexity of the disease. To work towards achieving this goal, we tested classification performance across different platforms. Overall correlations between samples across platforms using (fresh frozen and) (RNA microarray) and FFPE (RNA-Seq) tissues were over 0.5, as shown in Fig. S2. However, there were still samples with low correlation in ~25% of cases. To keep a greater number of samples, we applied filtering strategies to eliminate low correlated genes. Interestingly, the genes of EMT signatures showed the highest correlation compared to the MSI or TP53 signatures. So, the EMT signature was relevant to apply in cross-platform uses and could be applicable in the clinic. We could not discriminate the TP53-active and –inactive subtype accurately because it used only two genes (CDK1A and MDM2), and one of these two genes (MDM2) showed low correlation across two platforms, which is similar to our previous study using the nanostring platform with archival tissues^5^. Based on those results, appropriately sized signature gene sets are required for stable classifications to be applied across various platforms.

The clinical^4^ and molecular^3^ features of EMT have been well characterized and described. During the course of tumor progression, cancer cells with an epithelial origin frequently acquire a mesenchymal phenotype through EMT, one of the biological features promoting clinical heterogeneity in GC^6^. Recently, no remarkable benefits from adjuvant chemotherapy^6^ or checkpoint blockade^9^ have been observed among patients with EMT subtype GC. These observations demonstrate the importance of molecular subtypes of GC and an urgent need for the development of diagnostic methods to distinguish EMT subtypes in GC using archival tissue specimens for clinical applications.

For this purpose, we developed a mesenchymal subtype gene signature with the nanostring platform^5^. Given the disadvantages of NanoString, including its closed platform, high RNA quantity (usually 200 ng) required for the assay, and high cost for smaller sample sizes, we decided to develop the test with next-generation sequencing (NGS), which is currently being pursued at many institutions worldwide. Because of limited amounts of archival non-tumor tissue, we did not use non-tumor tissue for comparison.

With RNA-seq data, we could start experiments with as little as 10 ng of total RNA and obtained results from archival FFPE surgical specimens that correlated highly to those of FF tissue specimens. Moreover, we could decrease the number of genes used for molecular classifications compared to the original ones. Although we could not get 100% sensitivity or specificity, reasonable correlation was achieved for clinical application and the highest correlation was observed in the EMT signature. Moreover, we developed prediction scores with 10 genes to discriminate EMT subtypes with high R-squared values. Unexpectedly, we found that 6 out of 10 were already selected in our previous study with nanostring^5^, suggesting that results from those two different platforms significantly overlap and are reproducible.

In summary, we identified molecular subtypes of 50 GC tumors using FFPE RNA-Seq via the ACRG classification approach and filtered out genes with low Pearson correlation coefficients across two platforms. Finally, 55 or a minimum of 10 of 118 genes for EMT and 24 of 120 genes for MSI were selected from correlation computations. Given no remarkable benefits were seen from adjuvant chemotherapy or checkpoint blockade among GC patients with the EMT subtype, we are currently attempting to apply those genes to discriminate EMT subtypes of GC in a prospective clinical trial.

## Methods

The ACRG cohort consisted of 300 primary GC tissues from patients who underwent curative or palliative gastrectomy at Samsung Medical Center (SMC, Seoul, Korea) between 2004 and 2007 as previously reported^4^. The study protocol was reviewed and approved by the SMC Institutional Review Board (IRB No. 2010-12-088) and all methods were carried out in accordance with relevant guidelines and regulations. All participating subjects provided written informed consent and all patients were >18 years old. Clinical, pathological, surgical treatment and survival data were procured from review of electronic medical records. Out of 300 GC tissues, 50 tumor specimens were randomly selected for this transcriptome study using RNA from FFPE tissue specimens based on the tissue availability and molecular subtypes. For validation with different platforms, NanoString nCounter (NanoString Technologies, Seattle, WA) results in archival FFPE tissues from the same ACRG cohort (n = 71)^5^ were used.

### Sample preparation and RNA extraction

Total RNA was extracted from 2 to 4 sections of 4-µm thick FFPE sections from representative primary tumor blocks using the High Pure RNA Paraffin kit (Roche Diagnostic, Mannheim, Germany) after removing non-tumor elements by manual macrodissection guided by hematoxylin and eosin stained slides. Samples containing >50% tumor volume were used for this study.

After extraction, total RNA was quantified using a Qubit 2.0 Flourometer with the Broad Range RNA kit using the standard protocol. Samples containing <20 ng/μl total RNA were discarded and those containing 20 ng/μl or greater were selected for further transcriptome analyses.

### Data preparation and processing

Microarray gene expression profiles of GC patients were gathered from previous studies of the Affymetrix Human Genome U133plus 2.0 Array platform (GEO accession ID GSE62254)^4^. We extracted probe-level intensity signal values to gene expression profiles and assigned IDs from annotation information obtained from official gene symbols and the corresponding probe set. One gene expression was selected from the maximum signal value of the probe set.

Using the Ion Proton™ System, a matched gene expression profile was generated using an Ion AmpliSeq™ Transcriptome Human Gene Expression Kit (Thermo Fisher Scientific) designed to profile over 20,000 distinct human RNA targets using a highly multiplexed amplification method. Each amplicon represents a unique targeted gene and the average size of each amplicon was ~150 bp. Because of the targeted nature and small amplicon size, the total number of raw reads needed for DEG analysis for each library prepared with AmpliSeq is much smaller than typical whole-transcriptome RNA sequencing. For library preparation, a barcoded cDNA library was first generated using the SuperScript® VILO™ cDNA Synthesis kit from 10 ng of total RNA obtained from FFPE tissues. Then cDNA was amplified using Ion AmpliSeq™ technology to accurately maintain expression levels of all targeted genes as previously described^18^. Amplified cDNA Libraries were evaluated for quality and quantified using the Agilent Bioanalyzer High sensitivity chip. Libraries were then diluted to 100pM and pooled equally, with eight individual samples per pool. Pooled libraries were amplified using emulsion PCR on Ion Torrent OneTouch2 instruments (OT2) and enriched following the manufacturer’s instructions. Templated libraries were then sequenced on an Ion Torrent Proton™ sequencing system, using an Ion PI kit and chip V2^18^. After sequencing, gene transcripts per million (TPM) were calculated by ampliSeqRNA plugin (Thermo Fisher Scientific), where low-expressed genes with an average TPM less than 2 were excluded. Both the RNA-Seq and microarray data were transformed to log2 scale.

Before classifying molecular subtypes, we investigated gene and signature score reproducibility by calculating Pearson and Spearman correlation coefficients of each gene and signature score between RNA-Seq and Microarray samples. The GC gene expression signatures EMT, MSI, MSS/TP53+, and MSS/TP53− were referred from our previous ACRG study^4^. The scores for each signature were also calculated using ACRG study methods as the average log2-transformed expression values of all genes included in the respective signature. All statistical computations were performed using R v3.4.1 (R Core Team, Vienna, Austria; http://www.Rproject.org).

### Reproduction of molecular subtypes

We identified molecular subtypes using gene expression profiles of FFPE samples matched with ACRG. First, we identified outliers from high EMT and MSI signature scores and assigned them into cutoffs of MSS/EMT and MSI subtypes, respectively. Then, the remaining samples were classified into MSS/TP53+ and MSS/TP53− subtypes based on a cutoff determined by the Youden’s index of the ROC curve generated from TP53 signature scores for TP53 mutation. These samples were assigned into MSS/TP53+ subtypes if TP53 signatures scores were higher than the cutoff and otherwise were assigned into MSS/TP53− subtypes. The somatic mutation status was referred from the ACRG clinical information^4^. The ROC curve and the Youden’s index were estimated using the R package *pROC* v1.10.0.

### Classification performance

In order to assess the reproducibility of previous ACRG dataset subtype classification in FFPE samples, we computed sensitivity and specificity for each subtype, Cohen’s kappa, and overall accuracy with the R package *caret* v.6.0-77 using the subtypes identified in ACRG Microarray expression profile as a gold standard^4^. We also performed the ROC analysis and the Mann-Whitney *U* test on MSI and EMT signature scores and the MSI and EMT subtypes assigned in the ACRG cohort, respectively. For the ROC analysis, we dichotomized samples into MSI versus non-MSI and EMT versus non-EMT.

In addition, we replicated gene expression analyses carried out in the ACRG cohort using RNA-Seq along with clinical information. We estimated principal components from expression values of all genes available in the RNA-Seq data and calculated signatures scores of cell proliferation and cytokine signaling. The Spearman correlation coefficient was computed for pairs of gene expression signature scores and principal components. We used Kaplan-Meier survival curves to examine the association between overall survival and the ACRG subtypes identified with expression profiles of FFPE samples. Survival analysis was performed using the R package *GGally* v1.3.2 and *survival* v2.41-3.

### Development of prediction scores with 10 genes to discriminate EMT subtype

To develop a minimum number of gene sets to distinguish EMT subtype, we calculated the correlation between the EMT and gene expression level. We selected 10 gene sets that were highly correlated with EMT (correlation ≥ 0.65) in the RNA-seq data. After selection of gene sets, we constructed a prediction model with 10 genes using linear regression. The prediction model was evaluated by calculating sensitivity and specificity using the AUC package. For validation of this model, nanostring platforms from the ACRG (n = 71) and ARTIST (n = 72) cohorts and microarray platforms from the ACRG cohort (n = 300) were selected. All statistical analyses and visualized plots were performed in R program (R version 3.4.4).

## Supplementary information

Supplementary Figures
