# Supplementary material for: Reproduction of molecular subtypes of gastric adenocarcinoma by transcriptome sequencing of archival tissue
Source: Sci Rep. 2019 Jul 4;9:9675. doi: 10.1038/s41598-019-46216-6 (PMC6609684; doi:10.1038/s41598-019-46216-6)
Supplement: Supplementary file 1 — Supplementary Figures [file 41598_2019_46216_MOESM1_ESM.pdf]

Reproduction of molecular subtypes of gastric adenocarcinoma by transcriptome sequencing of archival tissue

You Jeong Heo[1], Charny Park[2,3], Doyeong Yu[3], Jeeyun Lee[4], Kyoung-Mee Kim[2]

[1]Department of Health Sciences and Technology, Samsung Advanced Institute for Health Sciences and Technology (SAIHST), Sungkyunkwan University and Samsung Medical Center, Seoul, Korea

[2]Department of Pathology and Translational Genomics, Samsung Medical Center, Sungkyunkwan University School of Medicine, Seoul, Korea

[3]Clinical Genome Analysis and Precision Medicine Branch, Research Institute, National Cancer Center, Goyang, Republic of Korea

[4]Department of Medicine, Division of Hematology-Oncology, Samsung Medical Center, Sungkyunkwan University School of Medicine, Seoul, Korea

Supplementary Figures

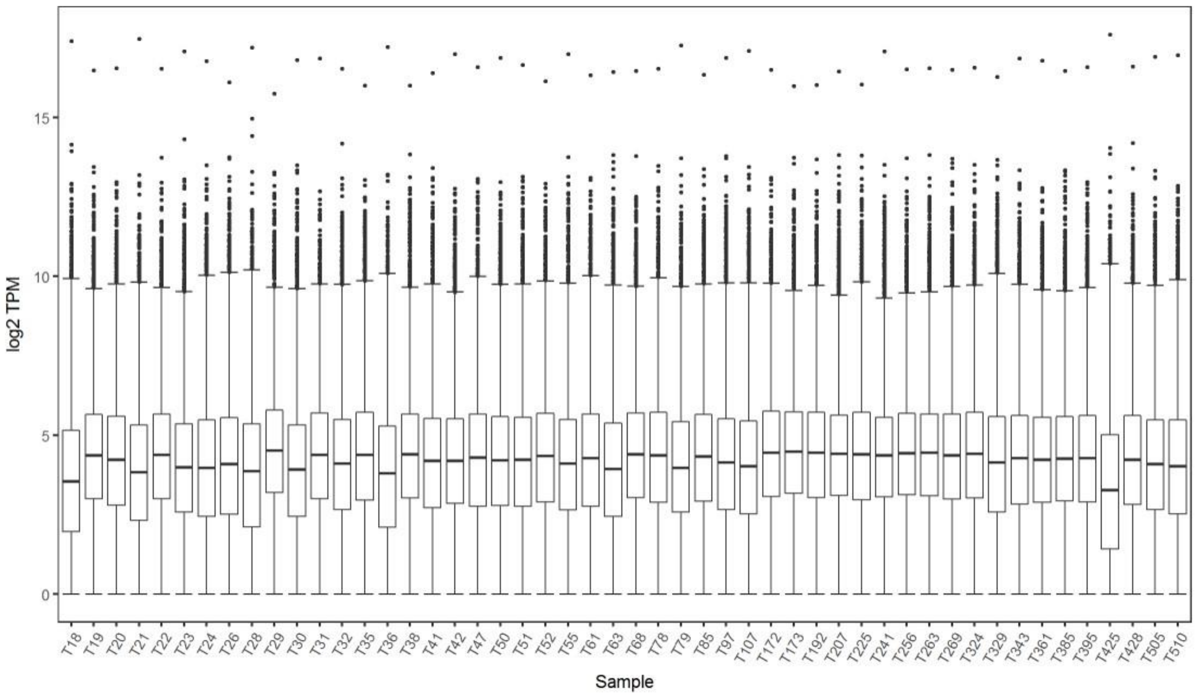

Supplementary Figure 1. Total gene expression profiles of FFPE samples showing similar distributions of log2 gene expression in 50 FFPE samples.

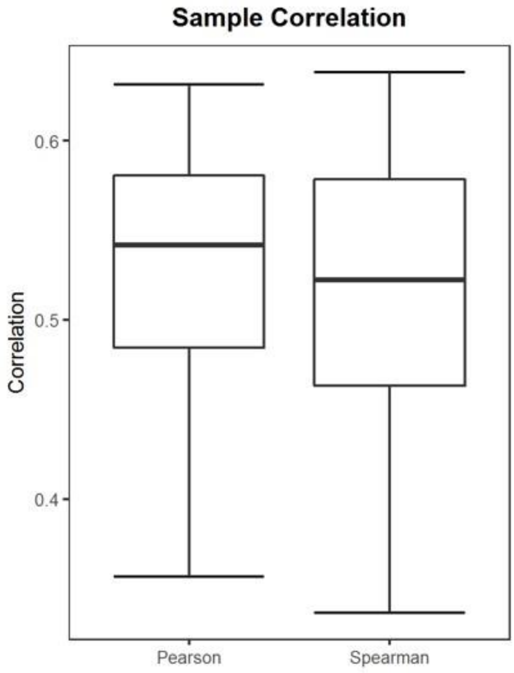

Supplementary Figure 2. Overall correlations between samples using fresh (RNA microarray) and archival (RNA-Seq) tissues by Pearson and Spearman.

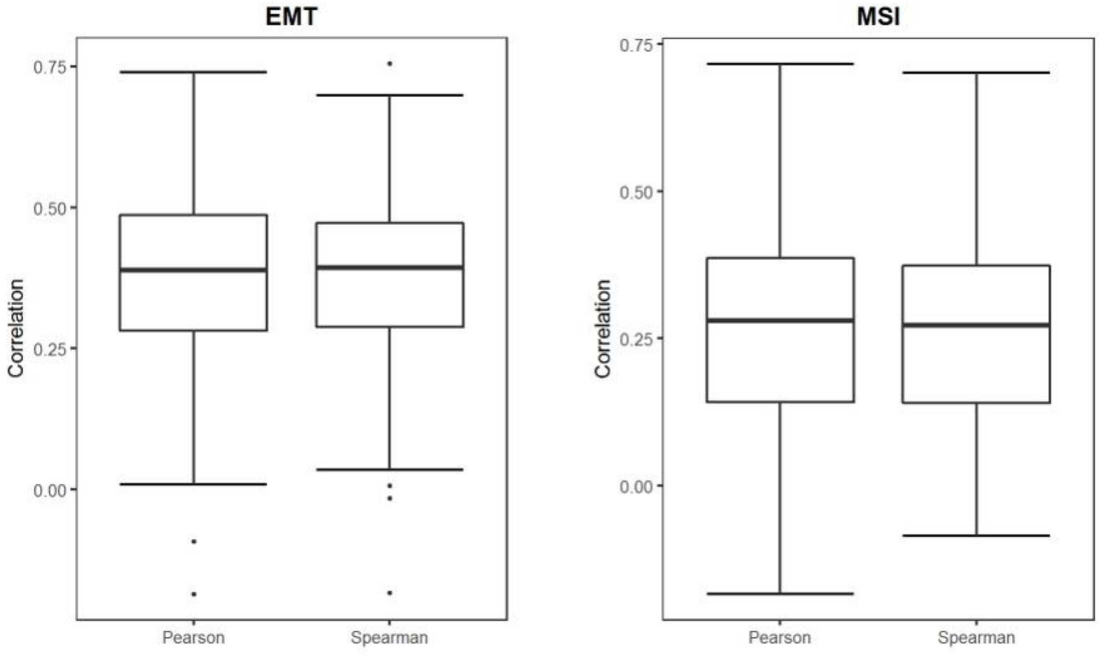

Supplementary Figure 3. The median Pearson correlations for genes in the EMT and MSI signatures by Pearson and Spearman.

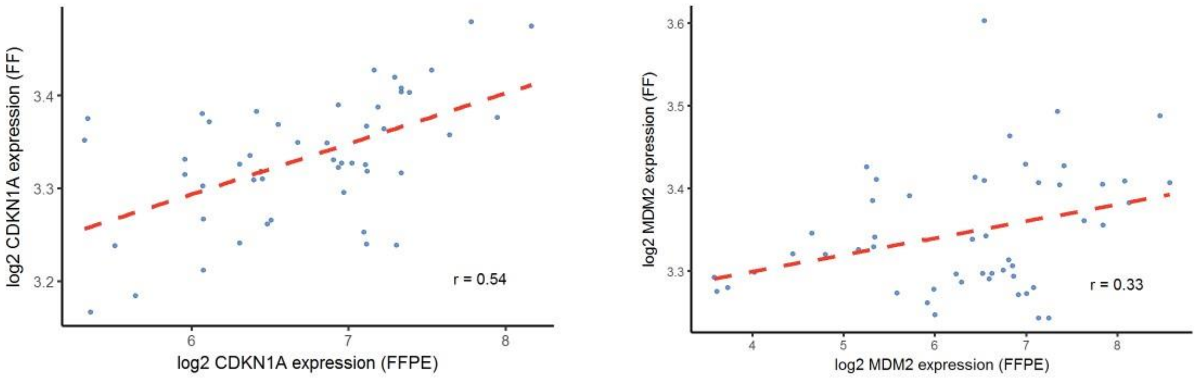

Supplementary Figure 4. The TP53 signature is composed of two genes, CDKN1A and MDM2, for which the Pearson correlations between two platforms were 0.54 and 0.33, respectively.

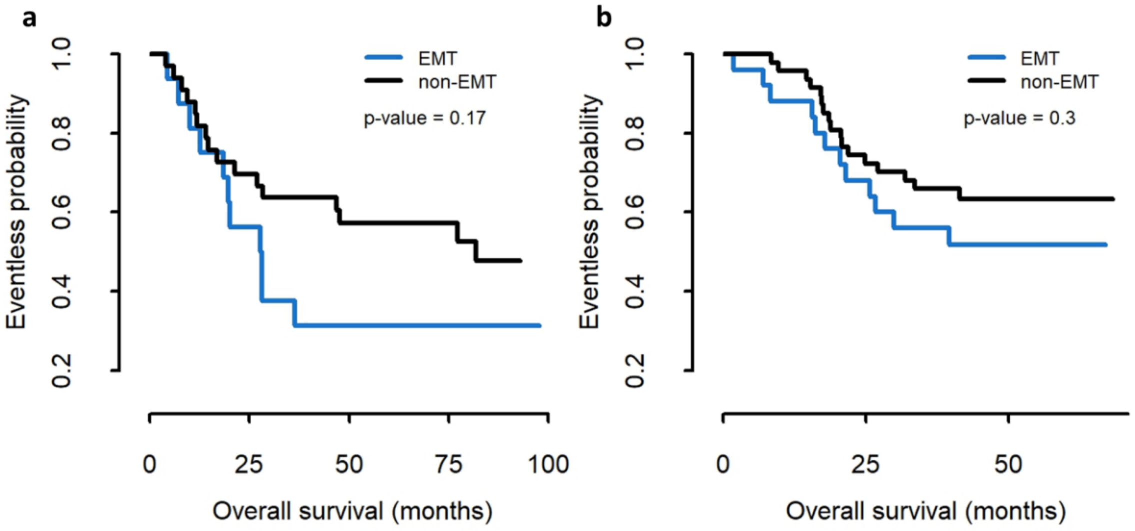

Supplementary Figure 5. Validation of EMT and non-EMT subtypes using nanostring platforms with FFPE tissues from the ACRG cohort (a) and ARTIST cohort (b).
